# A Dual-Path Computational Ghost Imaging Method Based on Convolutional Neural Networks

**DOI:** 10.3390/s24237869

**Published:** 2024-12-09

**Authors:** Hexiao Wang, Jianan Wu, Mingcong Wang, Yu Xia

**Affiliations:** 1 College of Computer Science and Technology, Changchun University, Changchun 130022, China; 221501490@mails.ccu.edu.cn (H.W.); 221501469@mails.ccu.edu.cn (M.W.); 241502526@mails.ccu.edu.cn (Y.X.); 2 Quantum Cryptography and Intelligent Network Security Laboratory, Changchun University, Changchun 130022, China

**Keywords:** computational ghost imaging, convolutional neural network, dual imaging, self-attention mechanism, image reconstruction

## Abstract

Ghost imaging is a technique for indirectly reconstructing images by utilizing the second-order or higher-order correlation properties of the light field, which exhibits a robust ability to resist interference. On the premise of ensuring the quality of the image, effectively broadening the imaging range can improve the practicality of the technology. In this paper, a dual-path computational ghost imaging method based on convolutional neural networks is proposed. By using the dual-path detection structure, a wider range of target image information can be obtained, and the imaging range can be expanded. In this paper, for the first time, we try to use the two-channel probe as the input of the convolutional neural network and successfully reconstruct the target image. In addition, the network model incorporates a self-attention mechanism, which can dynamically adjust the network focus and further improve the reconstruction efficiency. Simulation results show that the method is effective. The method in this paper can effectively broaden the imaging range and provide a new idea for the practical application of ghost imaging technology.

## 1. Introduction

Ghost imaging (GI), also known as correlation imaging, is a special quantum correlation imaging technology. Compared with traditional optical imaging, ghost imaging generates “ghost images” that cannot be seen by classical methods in a “non-local” way, that is, the images are generated on optical paths that do not contain objects [1]. By implementing light field fluctuation modulation and computational reconstruction, ghost imaging achieves not only greater efficiency in information acquisition but also enhanced flexibility in how image data are collected. Unlike traditional imaging methods, ghost imaging possesses unique capabilities, along with characteristics similar to ionized imaging [2].

The computational ghost imaging (CGI) scheme was first proposed in 2008, and Professor Shapiro’s team found that it is possible to calculate the measurement matrix of the signal branch, and only a barrel detector is required for imaging [3]. In 2009, the Bromberg team successfully implemented this solution [4], and CGI technology was born. The emergence of CGI brings a new perspective and possibility to the imaging field, which greatly reduces the complexity of the ghost imaging system. However, this method has some problems that affect its development. For example, the degree of downsampling is limited by the sparsity of the target [5], and the quality of the reconstructed image is highly sensitive to the detection noise [6]. In order to further improve image quality and reduce imaging time, scholars have proposed many experimental schemes successively: Compressed Sensing Ghost Imaging (CSGI), which can reduce the sampling rate [7,8]; by reconstructing the positive (negative) ghost image [9] through the beam fluctuation intensity, the corresponding ghost image [10] can reduce the imaging time. Normalized Ghost Imaging (NGI) with strong noise suppression performance [11]; Differential Ghost Imaging (DGI), which greatly improves image quality [12]; Temporal Ghost Imaging (TGI) of high-speed signals can be detected using low-speed detectors [13]. Through continuous improvement and innovation, ghost imaging technology has been rapidly developed, promoting its application in many fields such as communications, national defense, remote sensing, and military, especially in low light and harsh environments [14]. Its unique imaging method has shown broad application prospects in underwater detection, search and rescue and other aspects [15]. The above methods can effectively improve the imaging time and image quality of computational ghost imaging, but it still takes a long time to compute, and the image reconstructed at a low sampling rate has much noise and poor quality, which limits the development of computational ghost imaging technology.

Neural networks have strong learning ability and complex information processing ability, and more and more researchers have begun to pay attention to and explore the computational ghost imaging methods based on deep learning. In 2017, Situ Guohai combined deep learning technology with a ghost imaging scheme for the first time [16], constructed a simple deep neural network model, and used a large amount of data to train the model to reconstruct the target image. The experimental results proved that better reconstruction results were obtained at a 10% sampling rate. In 2019, Wang, F. et al., proposed a training method using simulation data [17]. Only the bucket detection value could be trained to reconstruct the target image. The amount of training data was greatly improved, and the way to obtain images became simpler and more diverse, and this method still has good effects on color image reconstruction [18]. With the continuous development of deep learning technology, more and more image enhancement neural networks are also applied to computational ghost imaging, such as recurrent neural network (RNN) [19]. Residual Neural Network (ResNet) [20], Generative Adversarial Networks (GANs) [21], The attention mechanism network [22], etc., realizes high-quality image reconstruction at a low sampling rate through the fusion of single or multiple network structures, and effectively improves imaging efficiency at the same time. These methods provide new ideas and directions for deep learning ghost imaging.

However, the existing ghost imaging methods based on deep learning are mainly based on the single optical path experiment scheme, using the single probe value to train the model, so the imaging range is limited to a certain extent. To expand the imaging range and enhance the practicality of ghost imaging technology, this paper presents a dual-path simulation imaging experiment scheme and introduces a dual-path computational ghost imaging approach utilizing a convolutional neural network. Different from the existing deep learning imaging methods, it is the first time to train the model by using the two-way probe as the input of the neural network. In addition, the self-attention mechanism is integrated into the network structure to dynamically adjust the focus of the network to achieve the accurate capture of image details and efficient reconstruction.

## 2. Materials and Methods

### 2.1. Computing Ghost Imaging Principles

**Figure 1:**
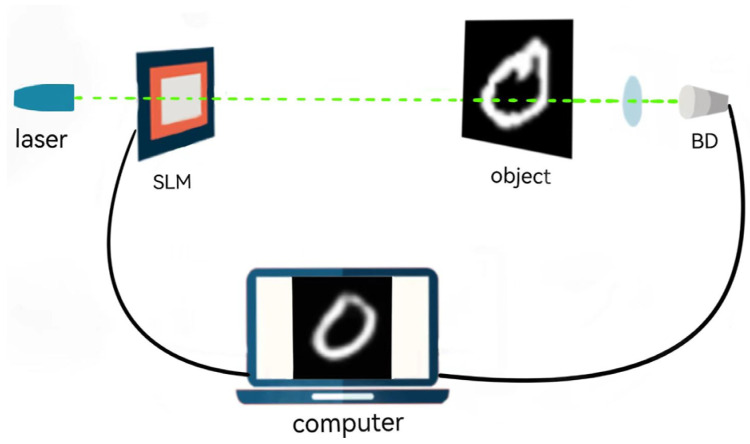
Schematic diagram of calculating ghost imaging.

Traditional ghost imaging requires two optical paths to reconstruct the image of the object, namely the reference optical path and the signal optical path [23]. Computational ghost imaging reduces the reference light path in it, and instead uses the spatial propagation distribution of the light source, using the spatial light modulator (SLM), the pseudo-thermal light field distribution generated by the SLM or Digital Micromirror Device (DMD) controls the modulation of the light field, so that only one detector and one beam are required to complete the image reconstruction. The schematic diagram of computational ghost imaging is shown in Figure 1.

In the figure, the light beam shines on the object through SLM, and a lens and a barrel detector (BD) without spatial resolution are placed behind the object to collect the total light intensity value reflected and projected after irradiating the object. The collected light intensity data can be associated with the known spatial distribution information to calculate the image of the object.

The second order intensity correlation function formula is used to reconstruct the image information of the object. Write the target object information as $O\left(x,y\right)$, The information of the speckle pattern obtained by SLM is denoted as $I^{N}(x,y)$, where (*x*,*y*) represents the horizontal and vertical coordinates of the object on the plane, N is the sampling number. From these two data operations, the bucket detection value $B^{N}$ can be obtained, expressed as

(1) $$B^{N}=\iint I^{N}(x,y)O(x,y)dxdy$$

The second-order correlation calculation formula for light intensity can be expressed as

(2) $$G^{2}(x,y)\;=\;<I^{N}(x,y)B^{N}>$$

In Formula (2), < > indicates that the sum is followed by the average, thus the reconstructed image can be obtained: $G_{GI}(x,y)$.

(3) $$G_{GI}(x,y)\;=\;<I^{N}(x,y)B^{N}>-<I^{N}(x,y)><B^{N}>$$

### 2.2. Dual Imaging Scheme

**Figure 2:**
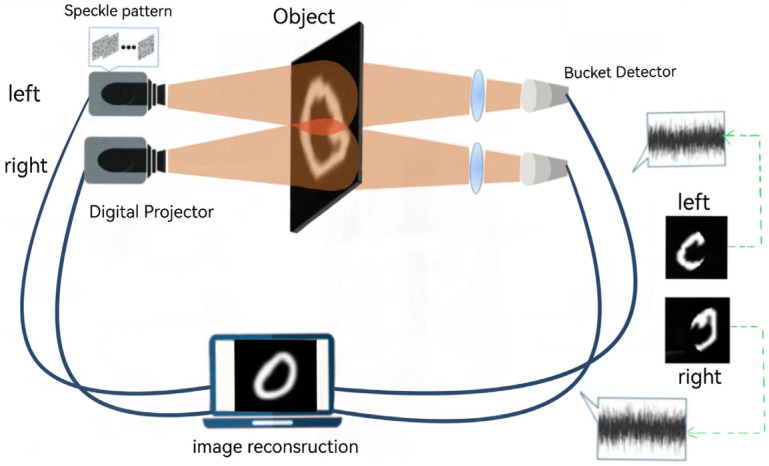
Schematic diagram of dual-channel imaging scheme device.

In this paper, a two-channel detection scheme is designed to broaden the imaging range. The scheme principle is shown in Figure 2.

In this scheme, two independent light source paths are set, two sets of identical random speckles are illuminated successively by a projector, and two lenses and two bucket detectors without spatial resolution are placed behind the target objects of the corresponding optical path, respectively, to collect N sets of bucket detection values collected by the corresponding optical path. In order to obtain the image of the target object completely, the two irradiation ranges (left and right) will overlap to a certain extent, so the two detection ranges will also overlap. Finally, the collected 2N group of bucket detection values will be input into the neural network to reconstruct the target image.

The neural network is trained by taking bucket detection values $B_{1}^{N}$, $B_{2}^{N}$, label *l* and corresponding target image $O\left(x,y\right)$ as input, takes the mean square error as the loss function, and reconstructs the image $G_{GI}(x,y)$ through the training output. Therefore, the mapping function of the model is expressed as

(4) $$G_{GI}\left(x,y\right)=f(B_{1}^{N},B_{2}^{N},l,\Theta )$$

The loss function L is expressed as

(5) $$L=\frac{1}{N}{\sum_{i=1}^{N}({G_{GI}\left(x,y\right)}_{i}-{O\left(x,y\right)}_{i})}^{2}$$

In order to optimize the model parameter Θ, the neural network learns the input-output mapping relationship by minimizing the loss function L to obtain the optimized model parameter $\Theta^{*}$, expressed as

(6) $$\Theta^{*}=arg\;\underset{\Theta }{\min}\frac{1}{N}\sum_{i=1}^{N}({{f(B_{1}^{N},B_{2}^{N},l,\Theta )}_{i}-{O\left(x,y\right)}_{i})}^{2}$$

The final reconstructed image is expressed as

(7) $$G_{GI}\left(x,y\right)=f(B_{1}^{N},B_{2}^{N},l,\Theta^{*})$$

## 3. Network Model

**Figure 3:**
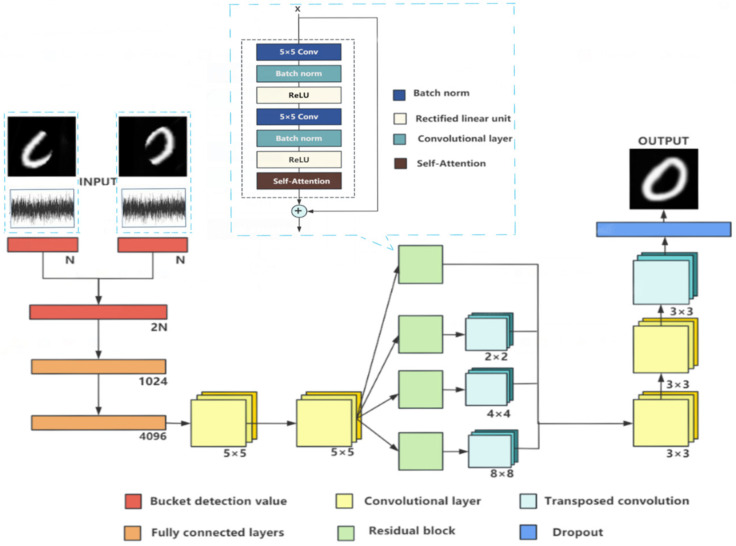
Network model diagram.

In this paper, a Dual Channel Computational Ghost Imaging (DCCGI) model is designed. By using two buckets of detection values and their corresponding target images as the input data set of the model, the neural network is iteratively optimized by using a large number of known images and their corresponding bucket signals as constraints. In each batch, the model calculates the output with forward propagation, then calculates the loss and performs backpropagation to update the model parameters, learning to extract features from it to produce an output close to the target image. The network model is shown in Figure 3.

First, the input double-channel bucket detection values are combined and then processed by the full connection layer to find the correlation between the input bucket signals. The dimension size of the input data is adjusted to 4096 under the function of the full connection layer, so that the input probe bucket value is mapped to a higher dimension representation, which provides convenience for the subsequent convolutional layer feature extraction. Features are deeply mined using two 5 × 5 convolutional layers, each focused on extracting richer and finer feature information from the data. In order to improve the stability and reliability of model training while effectively reducing overfitting [24], we successively introduce the batch normalization layer and dropout layer after each layer of convolution operation. Batch normalization ensures the stability of the output values at each layer, while dropout gives the model better generalization ability by randomly dropping some neuron connections. In addition, ReLU activation functions are used after each convolutional layer to preserve and map features in a nonlinear manner to enhance the modeling ability of the model for complex nonlinear data.

Loss function in a neural network is defined as the relationship between reconstructed images and known images. In order to further improve the model’s ability to capture multi-scale image features, the model also integrates residual blocks and self-attention mechanisms. These residual blocks realize cross-layer fusion and the enhancement of features through cross-layer connection, so that the model can simultaneously learn and retain image features of different scales. The residual module is mainly composed of a convolutional layer and a self-attention mechanism layer. First, the feature graph obtained after convolution is passed through two convolutional layers with a convolution kernel size of 5 × 5, and a batch normalization technique is used after each convolutional layer to ensure the stability of a feature distribution, and nonlinear transformation is introduced with a modified linear unit (ReLU) activation function. The feature representation is further enriched. The feature map then passes through the self-attention mechanism layer, which is able to effectively capture global dependencies and better recover the details and structure of the original image. The feature map processed by the self-attention mechanism is added to the original input feature map to form a residual connection. Through this design, the residual block can not only retain the key information of the image but also improve the performance and robustness of the model. In this model, due to the change in the size of the feature map caused by residual processing, the model adopts the transposed convolution layer for up-sampling operation to restore the original spatial size of the feature map. Then, two convolution layers with a 3 × 3 convolution kernel are introduced to deeply process the up-sampled feature maps to further extract and integrate feature information. These two convolutional layers not only enhance the representation of features, but also carry out the necessary refinement of features, and also help the model better capture subtle changes in the image. Ultimately, we reapply the transposed convolution layer to ensure that the final output image is restored to the desired size, resulting in the final output image. Furthermore, to enhance the model’s robustness and generalization capability while preventing overfitting during training, we incorporated a dropout layer at the model’s final stage. This layer helps mitigate the model’s over-dependence on the training data by randomly omitting some neurons, thereby improving the model’s generalization ability.

## 4. Results

### 4.1. Data Processing

The data set of this paper adopts the MNIST handwritten numerals dataset [25]. A total of 1500 pictures of the numbers 0 to 9 are selected as the training set and 375 as the test set. In order to simulate the data collected by the double-channel bucket detector and the wide-field imaging range, we first expanded the pixels of the original image from 28 × 28 to 64 × 64 and regarded it as an overall target image region to be reconstructed. A circular region with a radius of 23 pixels was taken as the target range for each route, and the pixels outside the region were modified to 0, and the pixel value of the target image remained unchanged. Bucket probe values are randomly generated with a binarization matrix based on a normal distribution as speckle and are generated by simulation calculation using MATLAB as an input of the neural network.

### 4.2. Experimental Results and Analysis

During the training process, the learning rate was set to 0.001, the batch size was established at 32, and the number of training epochs was fixed at 100. The loss function used was the Mean Squared Error (MSE) between the reconstructed image and the corresponding reference image, with model parameters updated using the gradient descent method.

**Figure 4:**
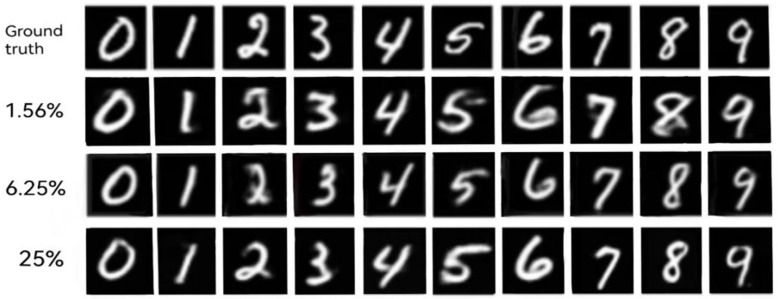
Simulation results.

In this paper, the full sampling rate is defined as 4096 (64 × 64) times of detection, aiming at three different sampling rates (1.56%, 6.25%, 25%). Figure 4 shows the display results of the reconstructed images of numbers 0 to 9 under three sampling rates.

As can be seen from the results of Figure 4, under the sampling rate of 1.56%, the scheme proposed in this paper is able to reconstruct the image close to the target, but the reconstruction result has obvious deformation, and some areas are fuzzy, but the overall figure can still be recognized. The numbers with more complex structures, such as “5”, “6”, and “8”, are deformed more severely than the numbers “1”, “4”, and “7”, which are composed of relatively simple strokes and only straight lines. When the sampling rate is increased to 6.25%, the results are significantly improved. When the sample rate is 25%, the digital structure is more similar and the details of the image are clearer.

**Figure 5:**
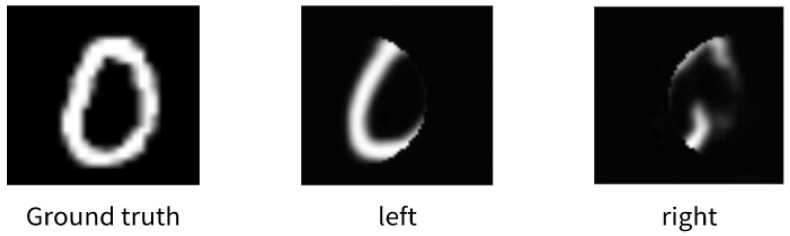
Results of single-channel imaging.

To illustrate the effectiveness of the proposed approach in increasing the imaging range, we employed the same network model structure for single-path imaging and compared the results to those obtained from the two-path imaging scheme. As depicted in Figure 5, the imaging area for the single path measures 1290.42 pixels, while the double path covers an area of 2047.79 pixels, with an overlap of 533.05 pixels. The imaging area of the double path is approximately 1.59 times larger than that of the single path, demonstrating a significant expansion of the imaging range with the two-path method.

**Figure 6:**
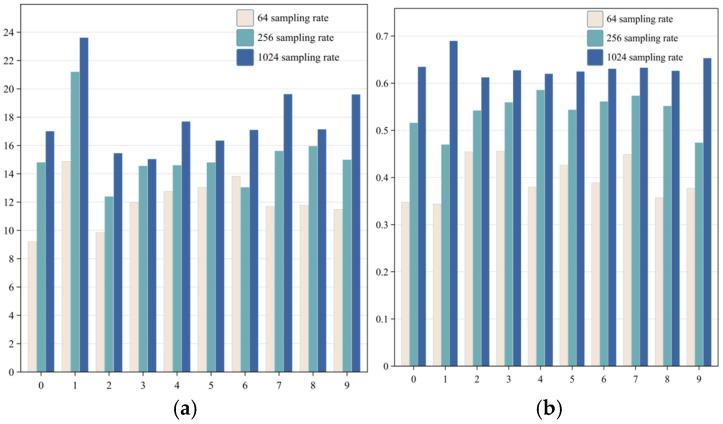
Comparison of PSNR and SSIM: (**a**) shows the PSNR values for images reconstructed with this method at various sampling rates; (**b**) presents the SSIM values for the images reconstructed with this method under different sampling rates.

In order to objectively evaluate the performance of this method, the quality of reconstructed images is analyzed using the Peak Signal-to-Noise Ratio (PSNR) and Structural Similarity Index (SSIM). As can be seen from the bar chart shown in Figure 6, as the sampling rate increases, the PSNR value and SSIM value of the reconstructed image also increase correspondingly, and numbers with relatively simple structures such as “1” and “7” change more significantly than other numbers. At the sampling rate of 6.25%, the highest PSNR value can reach 21.1944 db, and the highest SSIM value can reach 0.5856. Compared with the same figure at the sampling rate of 1.56%, the PSNR value and SSIM value are increased by 6.3118 db and 0.2059, respectively. The proposed method can also reconstruct the target image well at a lower sampling rate, and with the increase of the sampling rate, the reconstruction effect is better.

**Figure 7:**
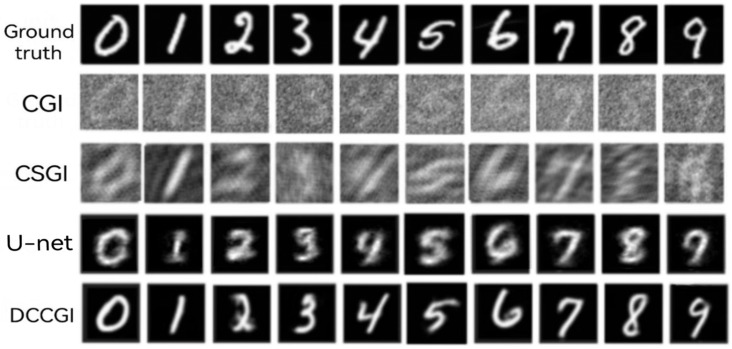
Comparison images of different imaging methods. Ground truth—target image; CGI—reconstructed images using traditional computational ghost imaging algorithms; CSGI—images reconstructed through compressive sensing algorithms; U-net—reconstructing images based on U-net neural network; dual channel—the reconstructed image of the model trained by the neural network designed in this scheme. The sampling rate is 6.25% for all samples.

In order to comprehensively and objectively demonstrate the reconstruction performance of our method, we used MATLAB to simulate three methods: traditional computational ghost imaging (CGI), compressive sensing ghost imaging (CSGI), and U-net based computational ghost imaging. Using the same target image and binary random speckle, we compared the reconstruction results with our method at a sampling rate of 6.52%. As shown in Figure 7, the imaging quality of our method is significantly higher than other methods. Although there is some degree of deformation, the digital structure of the reconstructed images is clearer compared to the other three methods.

Table 1 and Table 2 show the PSNR and SSIM values of reconstructed images using four different imaging methods at different sampling rates. It can be seen that as the sampling rate increases, the PSNR and SSIM values of each method also increase accordingly. The PSNR and SSIM values of our method are higher than those of other methods at all sampling rates.

**Figure 8:**
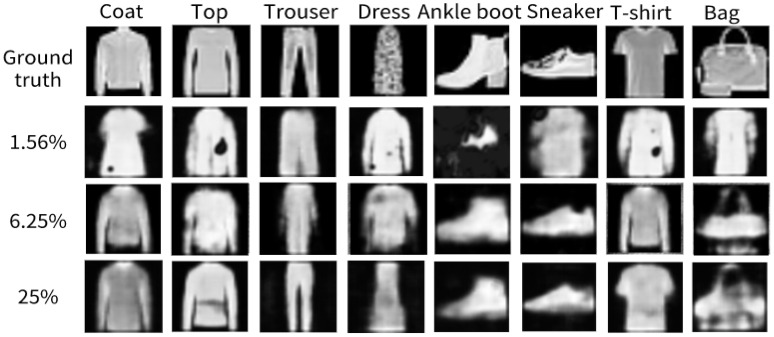
Simulation result of Fashion-MNIST.

In order to further verify the reconstructed image effect of the model in this paper under complex data sets, the Fashion-MNIST dataset was selected and tested at three sampling rates of 1.56%, 6.25%, and 25%. We selected part of the test results, as shown in Figure 8. At the sampling rate of 1.56%, the reconstruction results were poor, the reconstructed image was inconsistent with the target image, and the deformation was very serious. With the increase of the sampling rate to 6.25%, the results are improved, and the object contour can be basically reconstructed. When the sample rate reached 25%, the image details were more complete and clear, and there were fewer noise points, such as the gray value of the coat color, the waist part of the shirt, the heel of the naked boot, and the sleeve of the T-shirt.

**Figure 9:**
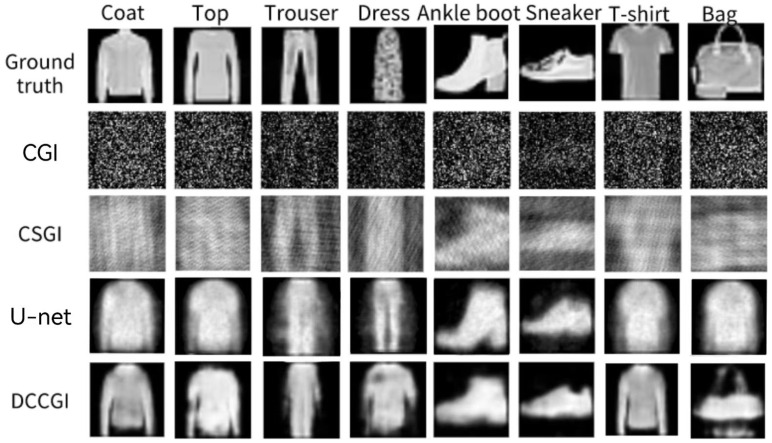
Comparison of simulation results of Fashion-MNIST.

In addition, we simulated CGI, CSGI, and U-net methods using MATLAB and compared the reconstruction results with our proposed method at a sampling rate of 6.52%. As shown in Figure 9, CGI cannot form images, and there is a lot of noise in the reconstructed image; CSGI pants and bare boots can distinguish object categories with the naked eye, but the deformation is extremely severe, and other categories cannot be distinguished; the U-net image is severely deformed and contains a large amount of noise. The reconstructed image in this scheme significantly reduces background noise, and the shapes and contours of objects are clearer, most of which can distinguish object categories.

### 4.3. Ablation Experiment

**Figure 10:**
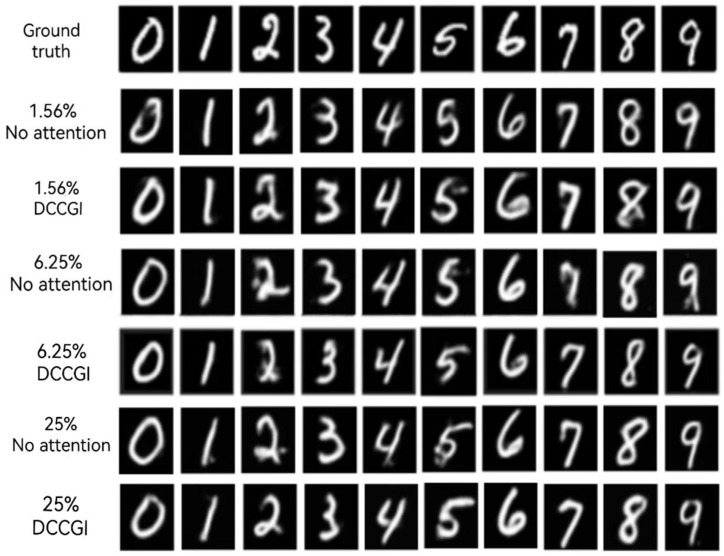
Imaging comparison of self-attention mechanism at different sampling rates.

To further verify the effectiveness of the proposed method, we conducted a detailed evaluation of the effect of adding self-attention mechanisms to the residual structure. Using the same data set for the image reconstruction task, we adopt the network model structure with the self-attention mechanism removed in the proposed method, and focus on the performance of the model under three different data compression rates (1.56%, 6.25%, and 25%). This is shown in Figure 10.

As can be seen from the figure, at the sampling rate of 1.56%, the presence or absence of an attention mechanism has little impact on the reconstruction results and there is no significant difference between them. With the increase of the sampling rate to 6.25%, compared with no attention mechanism, the image reconstructed with DCCGI has a smaller deformation and fewer noise points, such as the numbers “2”, “5”, “7”, and “9”. When the sampling rate is increased to 25%, compared with the non-attentional mechanism model, the background noise points of the reconstructed images are less, and the digital structure is closer to the target image.

Table 3 and Table 4 show the average PSNR and SSIM of reconstructed images with and without the attention mechanism across different sampling rates. The data indicate that at a sampling rate of 1.56%, there is little difference between the two methods; however, as the sampling rate increases, the disparity grows. With the attention mechanism in place, the PSNR and SSIM values of the reconstructed images are slightly higher than those without it. Specifically, at a sampling rate of 6.25%, the PSNR value is 2.9945 dB higher, and the SSIM value is 0.0468 greater. At a sampling rate of 25%, the PSNR increases by 2.2855 dB, and the SSIM increases by 0.0873. This analysis suggests that the residual structure incorporating the self-attention mechanism demonstrates significantly improved performance.

## 5. Conclusions

In order to obtain a wider range of target image information, this paper proposes a computational ghost imaging scheme with a dual path detection structure. Based on this structure, an image reconstruction network model based on a convolutional neural network and incorporating a self-attention mechanism is designed. Different from existing deep learning imaging methods, this paper attempts for the first time to use dual path detection values as inputs for convolutional neural networks, successfully reconstructing the target image. The integration of a self-attention mechanism further improves the reconstruction efficiency. We conducted simulation experiments using the MNIST handwritten digit dataset and the fashion style dataset, respectively, to verify the effectiveness of the model. In addition, the reconstruction quality of the image was tested and analyzed by combining PSNR and SSIM indicators. Finally, the rationality of the network structure design was demonstrated through ablation experiments. This article uses simulation methods for all experiments. Although the expected results have been achieved, this is only a preliminary theoretical verification and analysis. Further in-depth and detailed exploration and research are needed for broad applicability and practicality in real imaging scenarios.

## Figures and Tables

**Table 1:** PSNR values of different algorithms with different sampling rates.

Sampling Rate	CGI	CSGI	U-Net	Dual Channel
1.56%	5.4629	5.8777	9.7717	9.8328
6.25%	6.8658	7.6071	12.0825	15.3472
25%	8.2557	15.3226	15.8907	18.1141

**Table 2:** SSIM values of different algorithms with different sampling rates.

Sampling Rate	CGI	CSGI	U-Net	Dual Channel
1.56%	0.0252	0.0191	0.4208	0.4436
6.25%	0.0388	0.0949	0.4652	0.5290
25%	0.0708	0.2501	0.5229	0.6230

**Table 3:** Comparison of PSNR values with or without attention mechanism at different sampling rates.

Sampling Rate	Attentional Mechanism	No Attention Mechanism
1.56%	9.8328	9.3605
6.25%	15.3472	12.3527
25%	18.1141	15.8286

**Table 4:** Comparison of SSIM values with or without attention mechanism at different sampling rates.

Sampling Rate	Attentional Mechanism	No Attention Mechanism
1.56%	0.4436	0.4268
6.25%	0.5290	0.4822
25%	0.6230	0.5357

## Data Availability

The handwritten digit image dataset in this article can be accessed at http://yann.lecun.com/exdb/mnist and obtained from here (accessed on 4 March 2024). The clothing image dataset in this article, https://github.com/zalandoresearch/fashion-mnist, can be obtained from here (accessed on 31 March 2024).
